# Clinical study of a CT evaluation model combined with serum CA125 in predicting the treatment of newly diagnosed advanced epithelial ovarian cancer

**DOI:** 10.1186/s13048-018-0422-z

**Published:** 2018-06-18

**Authors:** Lu Qin, Huming Huang, Mengjie Chen, Yuejuan Liang, He Wang

**Affiliations:** Department of Gynecologic Oncology, Affiliated Tumor Hospital of Guangxi Medical University, 71 Hedi Road, Qingxiu District, Nanning, Guangxi 530021 People’s Republic of China

**Keywords:** CA125, Advanced epithelial ovarian cancer, Neoadjuvant chemotherapy, Debulking surgery

## Abstract

**Background:**

The treatment of newly diagnosed advanced epithelial ovarian cancer (EOC) was predicted by an ovarian cancer computed tomography (CT) evaluation model combined with serum CA125.

**Methods:**

Clinical data for 194 patients with advanced EOC treated with neoadjuvant chemotherapy (NACT) combined with interval debulking surgery (IDS) or primary debulking surgery (PDS) were retrospectively analyzed, and the appropriate treatment was predicted by comparing the subgroup differences in intraoperative situations, postoperative situations and survival rates.

**Results:**

There were no significant differences with respect to operation time, intraoperative blood loss, ideal tumor cytoreductive rate or postoperative complication rate between the NACT + IDS group and the PDS group with scores less than 5 (score < 5) (*p* = 0.764, *p* = 0.504, *p* = 0.906, *p* = 0.176). However, there was a statistically significant difference in overall survival rate between the two groups (*p* = 0.029), with better survival in the PDS group than in the NACT + IDS group. There were significant differences between the NACT + IDS group and the PDS group with scores greater than or equal to 5 (score ≥ 5). The former was better than the latter in terms of operation time, intraoperative blood loss, ideal tumor cytoreductive rate, and postoperative complication rate (*p* = 0.002, *p* = 0.040, *p* = 0.014, *p* = 0.021). However, there was no significant difference in overall survival rate between the two groups (*p* = 0.383).

**Conclusions:**

According to the new evaluation system, for a score < 5, we suggest that patients with newly diagnosed advanced EOC undergo PDS; for a score ≥ 5, we recommend NACT + IDS.

## Background

Ovarian cancer is one of the most common gynecological cancers and has the highest mortality of all gynecological cancers. Since ovarian cancer has no typical clinical manifestations or exact early screening methods, Siegel et al. [1] reported that 70% of ovarian cancer patients had advanced stage disease (stage III-IV) upon presentation and that the overall 5-year survival rate was only 27%. The prognosis of advanced ovarian cancer is closely related to the volume of postoperative residual tumor. Therefore, the ability to achieve ideal tumor cytoreduction becomes a pivotal prognostic factor for advanced ovarian cancer. The ideal tumor cytoreductive surgery standard is a maximum postoperative residual tumor diameter < 1 cm [2]. At present, there are two primary approaches to the treatment of advanced EOC: neoadjuvant chemotherapy combined with intermittent debulking surgery (NACT + IDS) and primary debulking surgery (PDS). To predict the treatment regimen of newly diagnosed EOC patients by an ovarian cancer CT evaluation model combined with serum CA125, clinical data for 194 newly diagnosed advanced EOC patients who came to our hospital from March 2010 to July 2016 were retrospectively analyzed.

## Methods

### Patient selection

Data were collected for 194 patients who had been newly diagnosed with advanced EOC when they arrived at our hospital from March 2010 to July 2016. According to our hospital gynecologists’ clinical experience, laboratory tests, imaging data and the patients’ own wishes, 99 patients with advanced EOC were treated with NACT + IDS. Patients were 29 to 75 years old (mean 53.05 ± 9.74 years); 78 cases were in pathological stage III, and 21 were in stage IV. Preoperative chemotherapy was based on platinum-based chemotherapy, and tumor cells were destroyed by chemotherapy. These patients completed 1~ 2 courses of platinum-based NACT before undergoing tumor cytoreductive surgery. Ninety-five patients with advanced EOC were treated with PDS. They were 25 to 79 years old (mean 54.72 ± 10.59 years old) with pathological stage III (79 cases) or stage IV (16 cases) disease. According to their wishes, 176 patients underwent platinum-based systemic vein NACT, and 18 patients had no postoperative chemotherapy.

### Measurement of CT evaluation model and CA125

We recorded the operation time, intraoperative blood loss, operative efficacy, postoperative recovery, postoperative complications, and postoperative follow-up of 194 patients with newly diagnosed advanced EOC. Based on the CT evaluation model of ovarian cancer patients that was established by Bristow et al. [3] in 2000, patients with a cumulative score ≥ 4 were not suitable for PDS. According to Vorgias et al. [4], when serum CA125 levels are higher than 500 U/ml, its accuracy is highest for predicting tumor cytoreductive surgery. Therefore, if at initial diagnosis the serum CA125 levels were ≥ 500 U/ml, we recorded 1 point. We now establish a New Scoring System based on a CT evaluation model combined with serum CA125 for patients with newly diagnosed advanced EOC. Five points will be designated as the demarcation point. One hundred ninety-four cases were divided into 4 groups by this New Scoring System: NACT + IDS group (score < 5, *n* = 49), PDS group (score < 5, *n* = 47), NACT + IDS group (score ≥ 5, *n* = 50) and PDS group (score ≥ 5, *n* = 48). In accordance with the intraoperative situation, postoperative situation and prognosis of patients in each group, appropriate treatment was predicted. The size of the residual tumor after surgery is an important prognostic factor. According to the 2014 National Comprehensive Cancer Network (NCCN) guidelines for ovarian cancer, non-ideal tumor cytoreductive surgery is indicated by postoperative residual ovarian cancer lesions ≥1 cm [5]. Four groups of patients were followed up until April 2017 (Table 1).

**Table 1:** New Scoring System based on a CT evaluation model combined with serum CA125

The initial diagnosis of serum CA125 levels (U/ml)	Point value
≥ 500	1
< 500	0
Predictive index parameter	Point value
Peritoneal thickening	2
Peritoneal implants ≥2 cm	2
Small bowel mesentery disease ≥2 cm	2
Large bowel mesentery disease ≥2 cm	2
Omentum extension to stomach, spleen, or lesser sac	2
Extension to pelvic sidewall, parametria, or hydroureter	2
Ascites-large volume (seen on all cuts)	2
Performance status ≥2	2
Suprarenal paraaortic lymph nodes ≥1 cm	2
Diaphragm or lung base disease ≥2 cm or confluent plaque	1
Inguinal canal disease or lymph nodes ≥2 cm	1
Liver lesion ≥2 cm on surface or any size parenchymal lesion	1
Porta hepatis or gallbladder fossa disease ≥1 cm	1
Infrarenal paraaortic lymph nodes ≥2 cm	1

### Statistical analysis

SPSS 20.0 software was used for statistical analysis. Normally distributed data are presented as the mean ± standard deviation (‾x ± S). Skewed data are presented as M (P_25_ ~ P_75_). The data were analyzed by *t*-test, χ^2^ test, non-parametric test or survival analysis. For all comparisons, differences were considered significant when *p* < 0.05. All findings were confirmed in at least one additional independent experiment.

## Results

### Clinicopathological factors in patients

After statistical analysis, patients in the NACT + IDS group (score < 5) were 53.16 ± 9.40 years old, and patients in the PDS group (score < 5) were 53.64 ± 10.79 years old. There was no significant difference in age between the two groups (*p* = 0.818). There was also no significant difference in clinical stage, pathological type or histological grade between the two groups (*p* = 0.343, *p* = 0.970, *p* = 0.598). Patients in the NACT + IDS group (score ≥ 5) were 52.94 ± 10.16 years old, and patients in the PDS group (score ≥ 5) group were 55.77 ± 10.39 years old. There was no significant difference in age between the two groups (*p* = 0.176). There was also no significant difference in clinical stage, pathological type or histological grade between the two groups (*p* = 0.876, *p* = 0.819, *p* = 0.448) (Table 2).

**Table 2:** Clinicopathological factors in the NACT + IDS and PDS groups of patients grouped according to the New Scoring System

Clinicopathological factors	Groups with score < 5			Groups with score ≥ 5		
	NACT + IDS	PDS	*p*	NACT + IDS	PDS	*p*
Age (y)	(53.16 ± 9.40)	(53.64 ± 10.79)	0.818	(52.94 ± 10.16)	(55.77 ± 10.39)	0.176
Stage						
III	38	40	0.343	40	39	0.876
IV	11	7		10	9	
Histology						
Serous	42	41	0.970	44	41	0.819
Mucous	6	5		5	5	
Endometrioid	1	1		1	2	
Grade						
G1	15	19	0.598	16	19	0.448
G2	4	3		3	5	
G3	30	25		31	24	

*NACT + IDS* neoadjuvant chemotherapy and interval debulking surgery, *PDS* primary debulking surgery

### Comparison of intraoperative and postoperative situations in the NACT + IDS and PDS groups (score < 5)

**Fig. 1:**
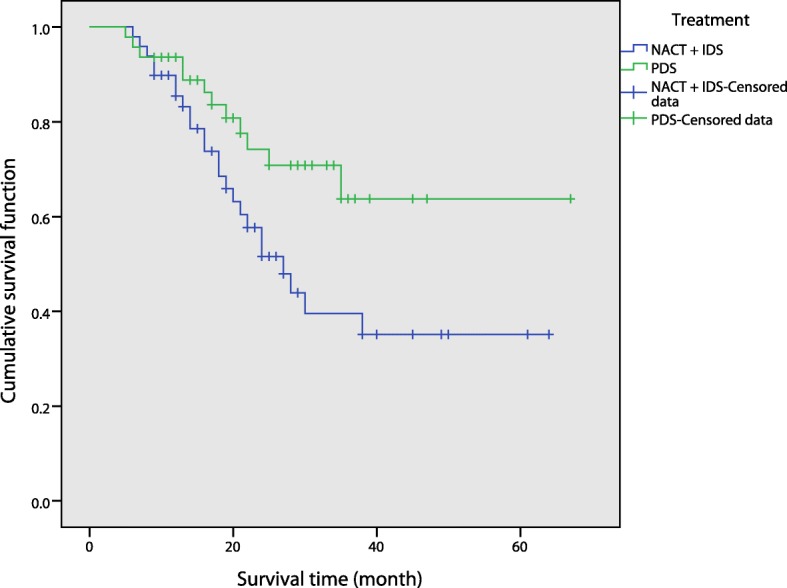
Overall survival rates were compared between the NACT + IDS and PDS groups with a score < 5

There were no significant differences in terms of operation time, intraoperative blood loss or ideal tumor cytoreductive rate between the two groups (*p* = 0.764, *p* = 0.504, *p* = 0.906). The incidence of complications in the NACT + IDS group was 12.24%, and that in the PDS group was 14.89%. There was no significant difference in the incidence of complications between the two groups after χ^2^ tests (*p* = 0.705). On follow-up of the NACT + IDS group, 20 patients survived, 24 died, and 5 were lost to follow-up. On follow-up of the PDS group, 28 patients survived, 12 died, and 7 were lost to follow-up. There was a significant difference in overall survival between the two groups (*p* = 0.029) (Table 3, Fig. 1).

**Table 3:** Surgery parameters in the NACT + IDS and PDS groups of patients with score < 5

Index	NACT + IDS	PDS	*p*
Number of cases	49	47	/
Operation time (min)	285.04 ± 92.24	278.57 ± 116.32	0.764
Intraoperative blood loss (ml)	550 (340~1300)	700 (300~1200)	0.504
Ideal tumor cytoreductive surgery	37	35	/
Ideal tumor cytoreductive rate (%)	75.51	74.47	0.906

### Comparison of intraoperative and postoperative situations in the NACT + IDS and PDS groups (score ≥ 5)

**Fig. 2:**
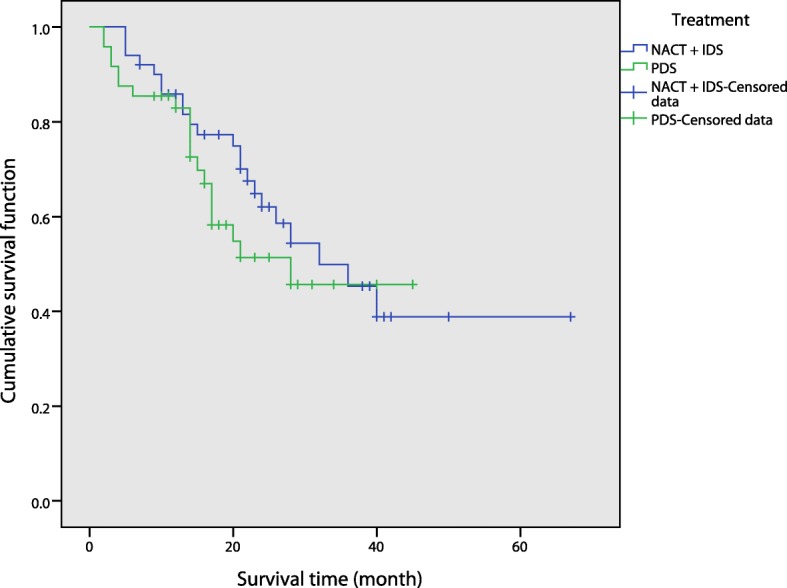
Overall survival rates were compared between the NACT + IDS and PDS groups with a score ≥ 5

There were significant differences in operation time, intraoperative blood loss and ideal tumor cytoreductive rate between the two groups (*p* = 0.002, *p* = 0.040, *p* = 0.014). That is, the operation time and intraoperative blood loss of patients in the NACT + IDS group were lower than those of patients in the PDS group. In addition, the ideal rate of reduction in NACT + IDS patients was higher than that it was in PDS patients. The incidence of complications was 4.00% in the NACT + IDS group and 18.75% in the PDS group. There was an obvious and significant difference in the incidence of complications between the two groups after χ^2^ tests (*p* = 0.021). We can conclude that the incidence of complications was lower in the NACT + IDS group than in the PDS group. On follow-up of the NACT + IDS group, 20 patients survived, 24 died, and 5 were lost to follow-up. On follow-up of the PDS group, 28 patients survived, 12 died, and 7 were lost to follow-up. There was no significant difference in overall survival between the two groups (*p* = 0.383) (Table 4, Fig. 2).

**Table 4:** Surgery parameters in the NACT + IDS and PDS groups of patients with score ≥ 5

Index	NACT + IDS	PDS	*p*
Number of cases	50	48	/
Operation time (min)	270.64 ± 92.64	339.48 ± 119.04	0.002
Intraoperative blood loss (ml)	550 (275~1100)	950 (400~1675)	0.040
Ideal tumor cytoreductive surgery	43	31	/
Ideal tumor cytoreductive rate (%)	86.00	64.58	0.014

### Comparative analysis of ideal tumor reduction at operation in the two PDS groups with scores < 5 and ≥ 5

According to case information, the two groups of patients with scores < 5 and ≥ 5 in terms of ideal tumor reduction were significantly different macroscopically. However, after statistical analysis, there was no statistically significant difference between the two groups of patients in terms of ideal tumor reduction rate (*p* = 0.296).

## Discussion

Ovarian cancer not only is a serious threat to the health and life of women but also has the highest mortality rate of all gynecological cancers. Because there is no exact method of screening, 70% of patients have reached the advanced stage by the time of presentation and often have abdominal metastasis, organ metastasis, ascites and pleural effusion. At present, the treatment of newly diagnosed patients with advanced epithelial ovarian cancer (EOC) is divided into two types: neoadjuvant chemotherapy combined with interval debulking surgery (NACT + IDS) and primary debulking surgery (PDS). NACT, also known as primary chemotherapy, refers to systemic chemotherapy that is administered before local treatment (such as radiotherapy or surgery). This treatment is primarily administered when PDS cannot achieve ideal tumor cytoreduction and/or patients are in poor condition and cannot tolerate tumor cytoreductive surgery. The ideal tumor cytoreductive rate can be improved by NACT [6]. Ovarian cancer is very sensitive to chemotherapy drugs. About 70 to 80% of patients with ovarian cancer have a complete response after chemotherapy [7]. Studies have shown that NACT has the following advantages: it kills metastastic cells surrounding the lesion, loosens the adhesion of the tumor and surrounding tissue, reduces tumor volume, improves the ideal tumor cytoreductive rate, and reduces the difficulty and complications of surgery [8]; it controls ascites and pleural effusion to improve the overall condition of patients and improve their surgical tolerance; it shortens the operation time, reduces intraoperative blood loss, and improves patient quality of life [9]; and it puts the tumor cells into a “dormant state”, reducing tumor spread and tumor cell seeding by surgery due to intraoperative squeezing and mechanical trauma stimulation, thereby reducing recurrence. However, the use of NACT for the treatment of patients with advanced ovarian cancer has been continuously controversial. For patients with advanced ovarian cancer who remain in good condition, studies have shown that PDS can achieve better tumor reduction and prognosis than NACT.

Based on the CT evaluation model of ovarian cancer patients that was established by Bristow et al. in 2000, patients with a cumulative score ≥ 4 (score ≥ 4) are not suitable for PDS. They noted that such patients should undergo NACT until the condition is under control prior to surgery. In addition, a large number of studies have shown that more than 85% of patients with ovarian cancer have a serum CA125 level higher than 35 U/mL, and serum CA125 levels are closely related to disease status in 93% of patients. Therefore, the level of serum CA125 is considered strong evidence in the evaluation of the ability of patients to achieve ideal tumor cytoreduction [10]. Vorgias et al. found that when serum CA125 levels were higher than 500 U/ml, the accuracy of its predictive ability was highest for patients undergoing tumor cytoreductive surgery to achieve the ideal standard, accounting for 84.2% in all observed ideal tumor cytoreductive surgeries. Obeidat et al. [11] supported this view and reported that a preoperative serum CA125 level of 500 U/ml was the most valuable indicator of whether ideal tumor cytoreductive surgery could be achieved. This indicator’s sensitivity and specificity were 72 and 73%, respectively. The positive predictive value was 68%. However, some investigators believed that the negative predictive value of CA125 is poor and that this measure cannot be used independently as an index to predict surgery. Therefore, in order to predict more suitable treatment for patients, we have assessed patients with newly diagnosed advanced EOC by taking advantage of an ovarian cancer CT evaluation model established by Bristow et al. combined with serum CA125 levels.

By comparing two groups (NACT + IDS and PDS), we found that there was no significant difference in the operation time, intraoperative blood loss, the ideal tumor cytoreductive rate or postoperative complications between the two groups of patients with a score < 5 (*p* = 0.764, *p* = 0.504, *p* = 0.906, *p* = 0.705). However, the overall survival rate was significantly different between the two groups (*p* = 0.029), with the PDS group having superior survival to the NACT + IDS group. This finding suggests that PDS did not show any advantage in terms of intraoperative and postoperative complications compared with the NACT + IDS group for patients with newly diagnosed advanced EOC when the score was < 5, but the postoperative quality of life and long-term prognosis of these patients were significantly better than those of the NACT + IDS group. For scores < 5, there was no significant difference between the NACT + IDS and PDS groups in terms of operation time, intraoperative blood loss and ideal tumor cytoreduction. Therefore, in order to avoid side effects and adverse reactions brought on by unnecessary chemotherapy, as well as excessive treatment costs and lengths of treatment that may delay the optimal operation time, we support the notion that advanced EOC patients should undergo PDS. In the two groups of patients with a score ≥ 5, the operation time of the NACT + IDS group was significantly shorter than that of the PDS group (*p* = 0.002). The amount of blood loss during operation in the former was less than that in the latter, and the ideal cytoreductive rate was higher (*p* = 0.040, *p* = 0.014). This finding showed that for scores ≥5, patients undergoing NACT could significantly improve their condition prior to surgery to reduce surgical difficulty and risks. In addition, the incidence of postoperative complications in the NACT + IDS group was significantly lower than that in the PDS group (*p* = 0.021). This result suggested that NACT could significantly reduce the incidence of complications and improve the quality of life of patients who were newly diagnosed with severe illness. None of these patients could undergo PDS immediately. In terms of overall survival rate, we found no significant difference between the two groups of patients (*p* = 0.383). NACT + IDS did not appear to improve prognosis. This result was in line with that of many published reports. Lozzi et al. [12] conducted a paired study of neoadjuvant chemotherapy with patients treated with standard treatment over the same period and showed no significant differences between the neoadjuvant chemotherapy group and the standard treatment group in terms of median survival time, median progression-free survival time and 3-year survival rate. Wright et al. [13] compared the efficacy of PDS and NACT + IDS in elderly patients with ovarian cancer using SEER data. They noted that the average survival of patients in the NACT + IDS and PDS groups was 15.8 months and 28.8 months, respectively, with 2-year survival rates of 36 and 56%, respectively. Fago-Olsen et al. [14] reported that the average overall survival of patients without residual tumor in the PDS group was better than that of those in the NACT + IDS group, at 55.5 and 36.7 months, respectively (*p* = 0.002). Simultaneously, patients in the NACT + IDS group had an increased risk of death after 2 years. We speculated that the reason for this analysis may be as follows: although NACT can reduce the difficulty and risk of surgery, the criterion for satisfactory tumor shrinkage in these studies was the same for patients with intermittent cytoreductive surgery following neoadjuvant chemotherapy as for direct cytoreductive surgery, namely, residual lesions less than 1 cm. The number of chemotherapy regimens after intermittent neoplastic cytoreductive surgery following neoadjuvant chemotherapy is fewer than the number after direct surgery, because neoadjuvant chemotherapy may increase the chemoresistance potential of ovarian cancer cells. However, NACT + IDS did not improve patient prognosis. By analyzing the ideal tumor cytoreductive rates of two PDS groups of patients who scored < 5 and ≥ 5, the difference in the ideal tumor cytoreductive rate of the two groups was approximately 10%, and the ideal surgical cytoreductive rate in patients in the PDS group and with a score < 5 was significantly higher than that of patients with a score ≥ 5. Although there was no statistically significant difference between the two groups (*p* = 0.296), we cannot conclude that there was no difference in the rate of reduction between the two groups. We believe the reason may stem from limitations in the collection of cases. For the treatment of patients with newly diagnosed advanced EOC, the choice of the re-established New Scoring System score cut-off point has substantial guiding significance. Whether there is a score < 5 or ≥ 5, patients with ovarian cancer can choose a relatively more favorable treatment. Therefore, we believe the choice of the cut-off point was reasonable. Taken together, we hypothesize that patients with newly diagnosed advanced EOC and score < 5 who can tolerate PDS at presentation may be better able to manage the optimal surgical treatment time, shorten the duration of patient treatment, reduce pain, reduce treatment costs and improve long-term outcomes. However, patients with a score ≥ 5 who were treated with NACT + IDS were more conducive to reducing the difficulty and risk of surgery, improving the reduction rate of tumor cells and improving the quality of life.

## Conclusion

Ovarian cancer has the highest mortality rate of all gynecological tumors. Most patients are already in advanced stages when they present for care. Advanced ovarian cancer tumor cells are widely disseminated. Their lesions are large and heavily infiltrate surrounding tissue. They also suppress organ function. Relevant reports show that only 42% of patients with severe disease can achieve ideal tumor reduction [15]. To delay the progression of the disease and to improve the quality of life, it is very important to choose treatment modalities for patients with newly diagnosed advanced epithelial ovarian cancer (EOC). We have evaluated the treatment by establishing a new evaluation system (new ovarian cancer CT model combined with serum CA125 levels). When patients are newly diagnosed with advanced EOC and have a score < 5, we suggest primary debulking surgery because this approach is able to save treatment time and costs and to improve the long-term prognosis. When the score is greater than or equal to 5 points, we are more inclined to recommend NACT + IDS in order to reduce the difficulty and risk of surgery and to improve the quality of life. There remains space for improvement and refinement of treatment options for patients with newly diagnosed EOC under the New Scoring System. Although at this stage of our research we can only provide some treatment ideas, we have laid a solid foundation for future research and analysis. There remain statistics from studies in other countries that show that NACT failed to improve 5-year survival rate and median survival time in patients with advanced ovarian cancer [16]. Therefore, in the future, we also need to explore and verify the treatment modalities of NACT and to conduct additional research and analysis on the New Scoring System to further demonstrate the rationale and validity of the scoring system and to provide strong evidence to support the choice of treatment modality.

## Abbreviations

EOC: Epithelial ovarian cancer
IDS: Interval debulking surgery
NACT: Neoadjuvant chemotherapy
PDS: Primary debulking surgery
